# Supplementary material for: Cancer cell-expressed BTNL2 facilitates tumour immune escape via engagement with IL-17A-producing γδ T cells
Source: Nat Commun. 2022 Jan 11;13:231. doi: 10.1038/s41467-021-27936-8 (PMC8752682; doi:10.1038/s41467-021-27936-8)
Supplement: Supplementary file 1 — Supplementary Information [file 41467_2021_27936_MOESM1_ESM.pdf]

Supplementary information

Cancer cell-expressed BTNL2 facilitates tumour immune escape via engagement with IL-17A-producing γδ T cells

Yanyun Du[1,#], Qianwen Peng[1,#], Du Cheng[2], Ting Pan[1], Wanwei Sun[1], Heping Wang[1], Xiaojian Ma[1], Ruirui He[1,3,4], Huazhi Zhang[1], Zhihui Cui[1], Xiong Feng[1], Zhiqiang Liu[1], Tianxin Zhao[1], Wenjun Hu[1], Lei Shen[2], Wenyang Jiang[5], Na Gao[6], Bradley N. Martin[7], Cun-Jin Zhang[8], Zhanguo Zhang[9], Chenhui Wang[1,3,4*]

1 Key Laboratory of Molecular Biophysics of the Ministry of Education, National Engineering Research Center for Nanomedicine, College of Life Science and Technology, Huazhong University of Science and Technology, Wuhan 430074, China.
2 Department of Gastroenterology, Renmin Hospital of Wuhan University, Wuhan, China.
3 The Key Laboratory for Human Disease Gene Study of Sichuan Province and the Department of Laboratory Medicine, Sichuan Provincial People's Hospital, University of Electronic Science and Technology of China, Chengdu 611731, China.
4 Research Unit for Blindness Prevention of the Chinese Academy of Medical Sciences (2019RU026), Sichuan Academy of Medical Sciences and Sichuan Provincial People's Hospital, Chengdu, Sichuan, China.
5 Department of Thoracic Surgery, Renmin Hospital of Wuhan University, Wuhan, China.
6 Key Laboratory of Molecular Biophysics of the Ministry of Education, Hubei Key Laboratory of Bioinformatics and Molecular-imaging, Department of Bioinformatics and Systems Biology, College of Life Science and Technology, Huazhong University of Science and Technology, Wuhan 430074, China.
7 Division of pulmonary and critical care medicine, Brigham and Women’s Hospital, Harvard Medical School, Boston, MA, USA.
8 Department of Neurology of Drum Tower Hospital, Medical School and the State Key Laboratory of Pharmaceutical Biotechnology, Nanjing University, Nanjing, Jiangsu, China.
9 Department of Hepatic Surgery Center, Tongji Hospital, Tongji Medical College, Huazhong University of Science and Technology, Wuhan 430070, China.

# These authors contributed equally: Yanyun Du and Qianwen Peng

* Correspondence: Chenhui Wang (wangchenhui@hust.edu.cn)

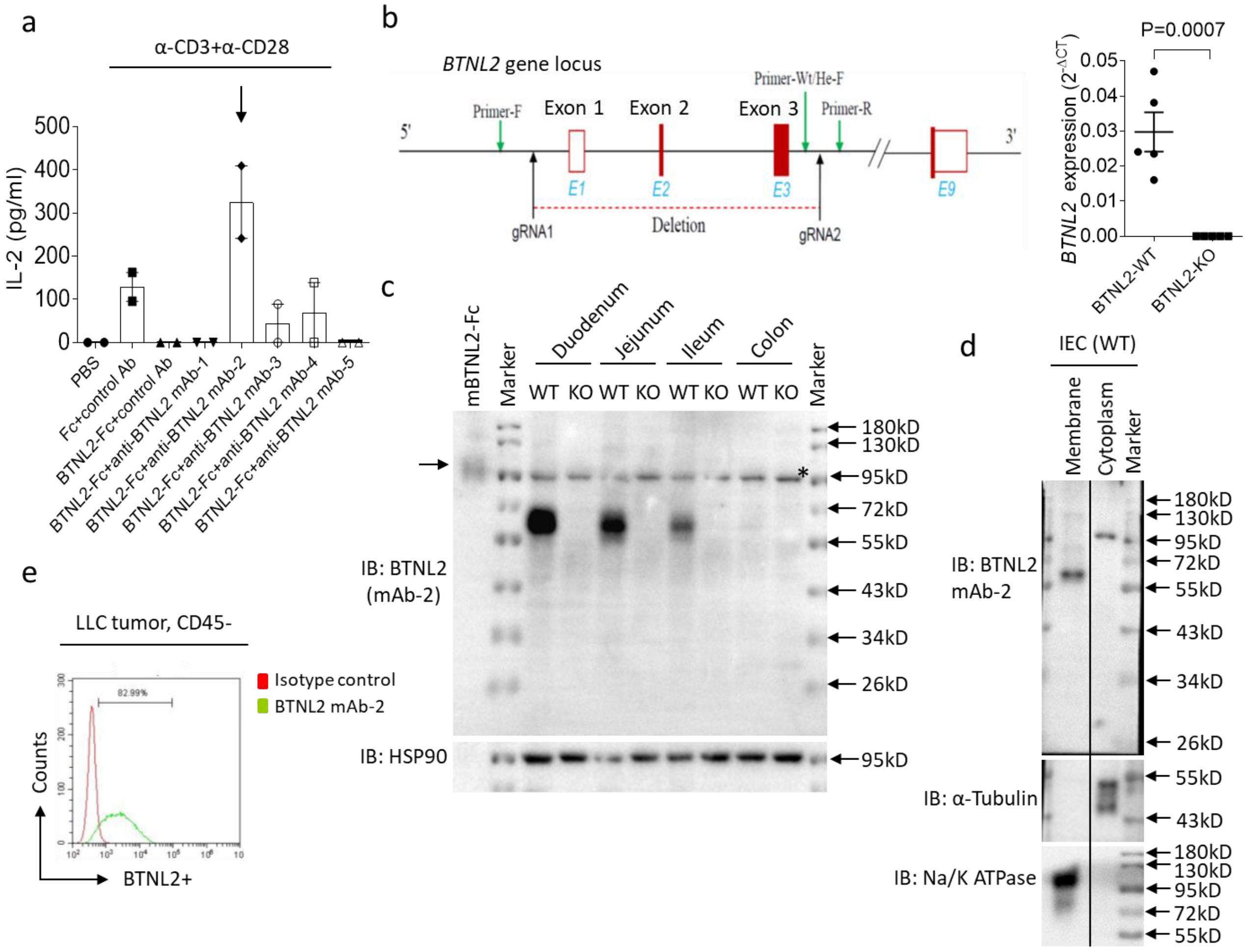

Supplementary Figure 1 Screening of BTNL2 blocking monoclonal antibody. (a) $CD4^+$ T cells were isolated from mice spleen by $CD4^+$ T Cell Isolation Kit, and were stimulated as indicated in the Materials and methods. 2 days after stimulation, supernatant was harvested and examined for IL-2 production. Arrow indicates the mAb-2 chosen for the following experiments (supernatant of hybridomas were used in this experiment). Fc represents recombinant IgG Fc region alone as a control. (**b**) The design strategy of BTNL2-KO mice was shown, and depletion was confirmed by real-time PCR (right panel, P=0.0007). **(c)** Western blot analysis of BTNL2 expression by using BTNL2 mAb-2 in different intestinal tissues from littermate control or BTNL2-KO mice (arrow indicates the band of BTNL2-Fc, and asterisk indicates a non-specific band) (1μg/ml of anti-BTNL2 mAb was used). **(d)** Small intestinal epithelial cells (IECs) were isolated from littermate control or BTNL2-KO mice, followed by membrane-cytoplasm isolation described in the Methods, and BTNL2 expression by using BTNL2 mAb-2 was analyzed in the membrane or cytoplasmic fractions (1μg/ml of anti-BTNL2 mAb was used). **(e)** Flow cytometry analysis of BTNL2 expression in LLC tumour was performed by using BTNL2 mAb-2 (2μg/ml of anti-BTNL2 mAb was used for flow cytometry analysis). All data are mean±s.e.m. *P<0.05, **P < 0.01, ***P < 0.001, ****P < 0.0001 based on two-tailed unpaired T test for **(b)**. Data in **(a-e)** are representative of three independent experiments.

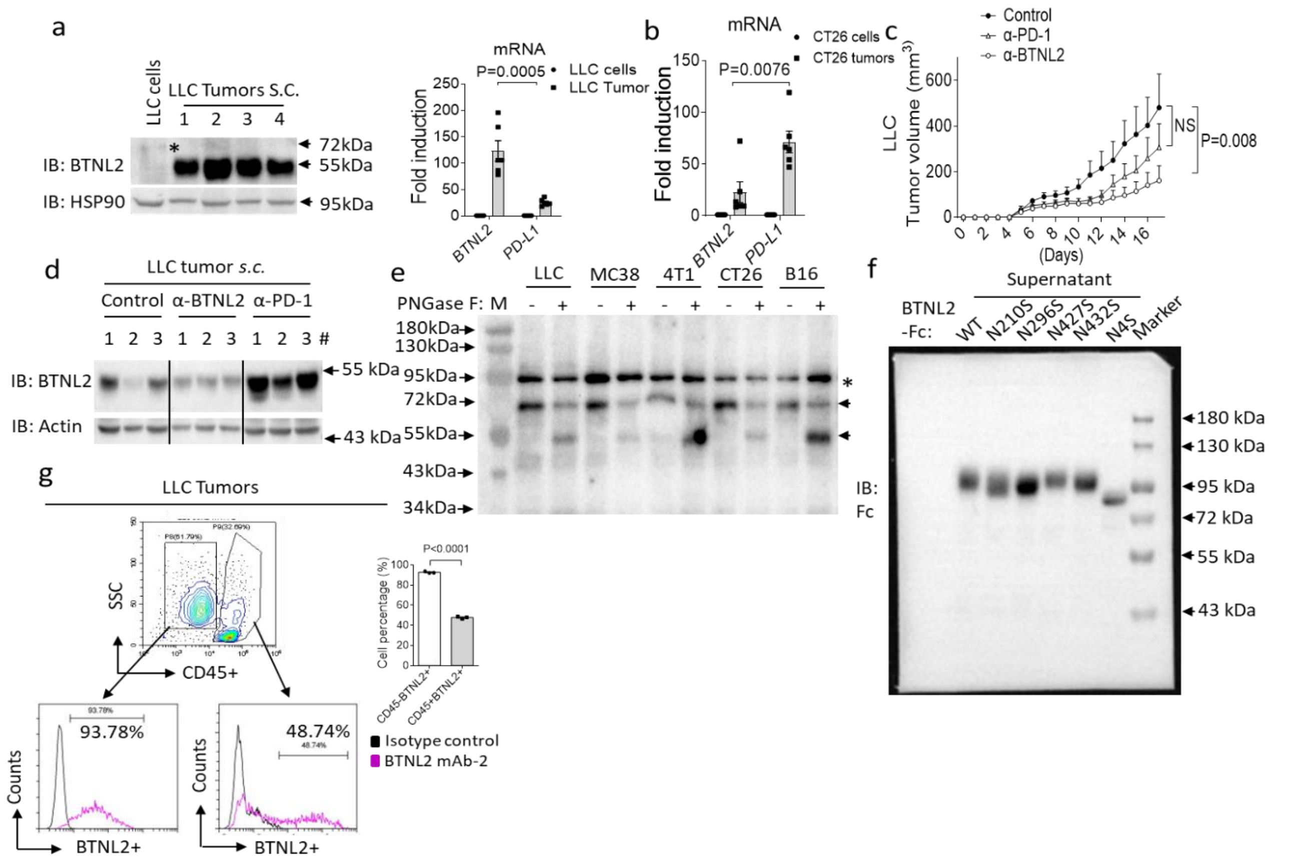

Supplementary Figure 2 Unglycosylated BTNL2 protein was highly induced in the TME. (a) Lysates from LLC cells and LLC tumours (left panel) were analyzed by western blot by using BTNL2 mAb-2 (1 µg/ml for western blot), and probed for the indicated proteins (asterisk indicates 72kDa band of BTNL2). *BTNL2* and *PD-L1* mRNA expression from LLC cells and LLC tumours were shown on the right (n=6, P=0.0005 for *BTNL2* vs *PD-L1* mRNA expression from LLC tumours). **(b)** *BTNL2* and *PD-L1* mRNA expression from CT26 cells and CT26 tumours were shown (n=6, P=0.0076 for *BTNL2* vs *PD-L1* mRNA expression from CT26 tumours). **(c)** Primary LLC tumour growth kinetics of mice after intraperitoneal injection of isotype control Ab, anti-BTNL2 or anti-PD-1 mAb was shown (n=6 for each group, P=0.008 for control vs α-BTNL2, NS for control vs α-PD-1). **(d)** Lysates from LLC tumours treated as indicated were analyzed by western blot by using BTNL2 mAb-2 (1µg/ml for western blot), and probed for the indicated proteins. **(e)** Five different cell lines were left untreated or treated with PNGase F for 4 hours, followed by western blot analysis of BTNL2 protein expression by using BTNL2 mAb-2 (asterisk indicates a non-specific band) (1µg/ml of mAb-2 for western blot). **(f)** Different BTNL2-Fc constructs were transfected into 293F cells, and 6 days after transfection, culture media was collected and concentrated, followed by western blot analysis of indicated proteins by using Fc antibody. "N4S" represents the quadruple mutant of BTNL2 in which all the four asparagine were replaced to serine. **(g)** BTNL2 expression in LLC tumour were analyzed by flow cytometry (2µg/ml of mAb-2 was used for flow cytometry analysis), and quantification was shown on the right panels (n=3, P<0.0001). All data are mean±s.e.m. *P<0.05, **P < 0.01, ***P < 0.001, ****P < 0.0001 based on two-sided unpaired T test for **(a** and **b)** and Two-way ANOVA for **(c)**. Data in **(a-f)** are representative of three independent experiments.

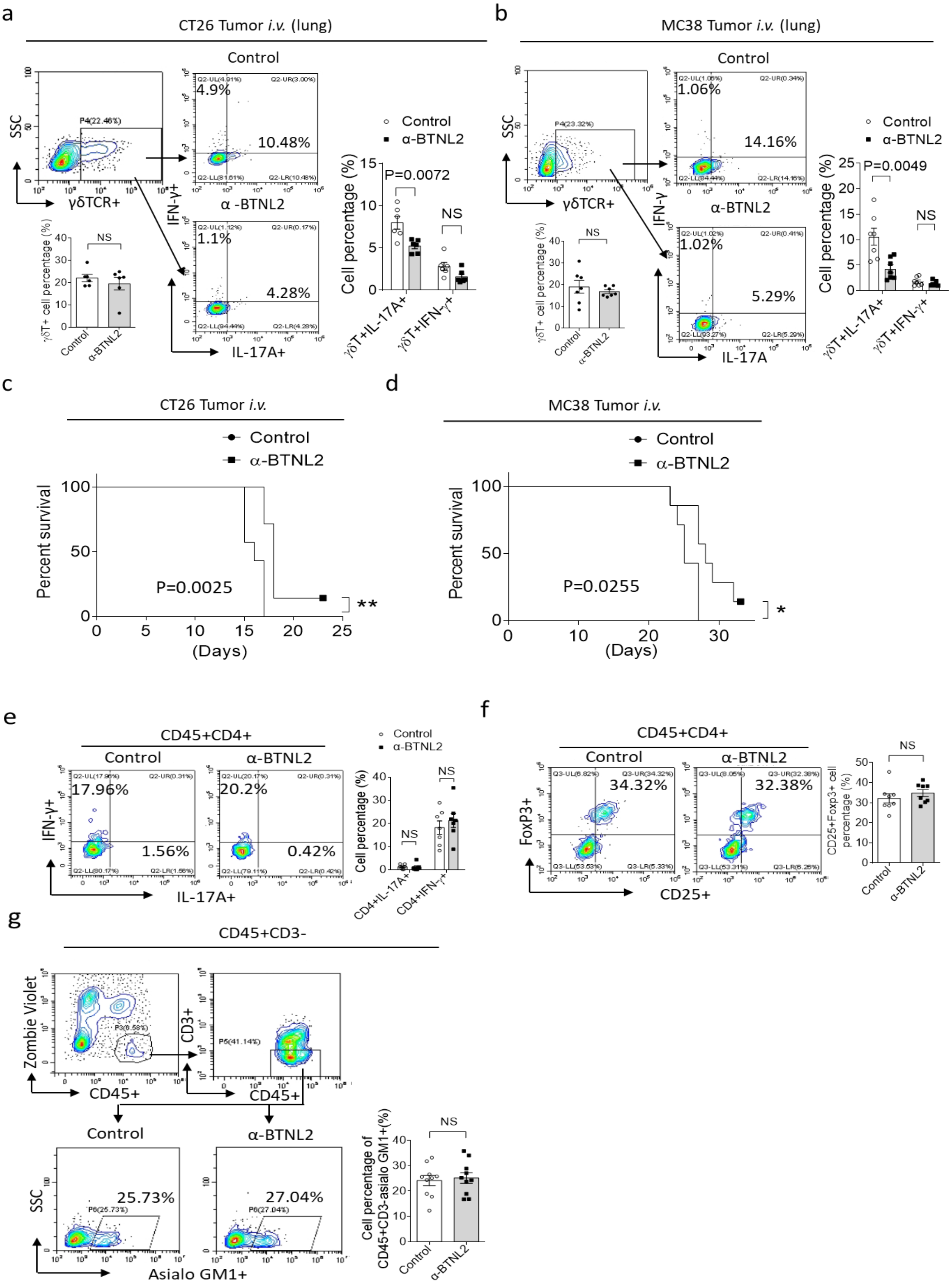
a
CT26 Tumor i.v. (lung)
Control
SSC
P4(22.46%)
γδTCR+
IFN-γ+
4.9%
10.48%
α -BTNL2
1.1%
4.28%
IL-17A+
γδT+ cell percentage (%)
NS
Control
α-BTNL2
Cell percentage (%)
P=0.0072
NS
γδT+IL-17A+
γδT+IFN-γ+
b
MC38 Tumor i.v. (lung)
Control
P4(23.32%)
1.06%
14.16%
α-BTNL2
1.02%
5.29%
IFN-γ
IL-17A
P=0.0049
c
CT26 Tumor i.v.
Control
α-BTNL2
Percent survival
P=0.0025
**
(Days)
d
MC38 Tumor i.v.
P=0.0255
*
e
CD45+CD4+
Control
α-BTNL2
17.96%
1.56%
20.2%
0.42%
CD4+IL-17A+
CD4+IFN-γ+
f
CD45+CD4+
FoxP3+
34.32%
32.38%
CD25+
CD25+Foxp3+ cell percentage (%)
g
CD45+CD3-
Zombie Violet
P3(6.58%)
CD45+
CD3+
P5(41.14%)
25.73%
27.04%
Asialo GM1+
Cell percentage of CD45+CD3-asialo GM1+(%)

Supplementary Figure 3 Anti-BTNL2 mAb has therapeutic effect for intravenous injection-induced tumour. (a-b) Mice were intravenous injected 2×10^5 of CT26 **(a)** or 2×10^5 of MC38 **(b)** tumour cells, and after control Ab or anti-BTNL2 mAb treatment (200µg/mouse), infiltrated cells from lung of CT26 cells **(a)** or MC38 cells intravenously-injected **(b)** mice were isolated, followed by flow cytometry analysis as indicated (**a**, n=7, P=0.0072 for $\gamma\delta T^+$IL-17A$^+$ cell Percentage, NS for $\gamma\delta T^+$IFN-γ^+ cell Percentage, and **b**, n=7 P=0.0049 for $\gamma\delta T^+$IL-17A$^+$ cell Percentage, NS for $\gamma\delta T^+$IFN-γ^+ cell Percentage). **(c-d)** Mice were intravenous injected 2×10^5 of CT26 **(c)** or 2×10^5 of MC38 **(d)** tumour cells, followed by intraperitoneal injection of control or anti-BTNL2 mAb as described in the Materials and methods (200µg/mouse of control or mAb-2) (**c**, n=7, P=0.0025, and **d**, n=7, P=0.0255). Mice survival was shown. **(e-g)** Infiltrated cells in CT26 subcutaneous tumour (18 days after tumour implantation) were analyzed by flow cytometry (n=7 for each group, NS for CD4$^+$IL-17A$^+$, CD4$^+$IFN-γ^+, CD25$^+$Foxp3$^+$, CD45$^+$CD3$^-$asialo GM1$^+$cell Percentage from control vs α-BTNL2). All data are mean±s.e.m. *P<0.05, **P < 0.01, ***P < 0.001, ****P < 0.0001 based on two-tailed unpaired T test for **(a, b, e-g)**, Log-rank (Mantel-Cox) Test for **(c** and **d)**. Data are representative of three independent experiments.

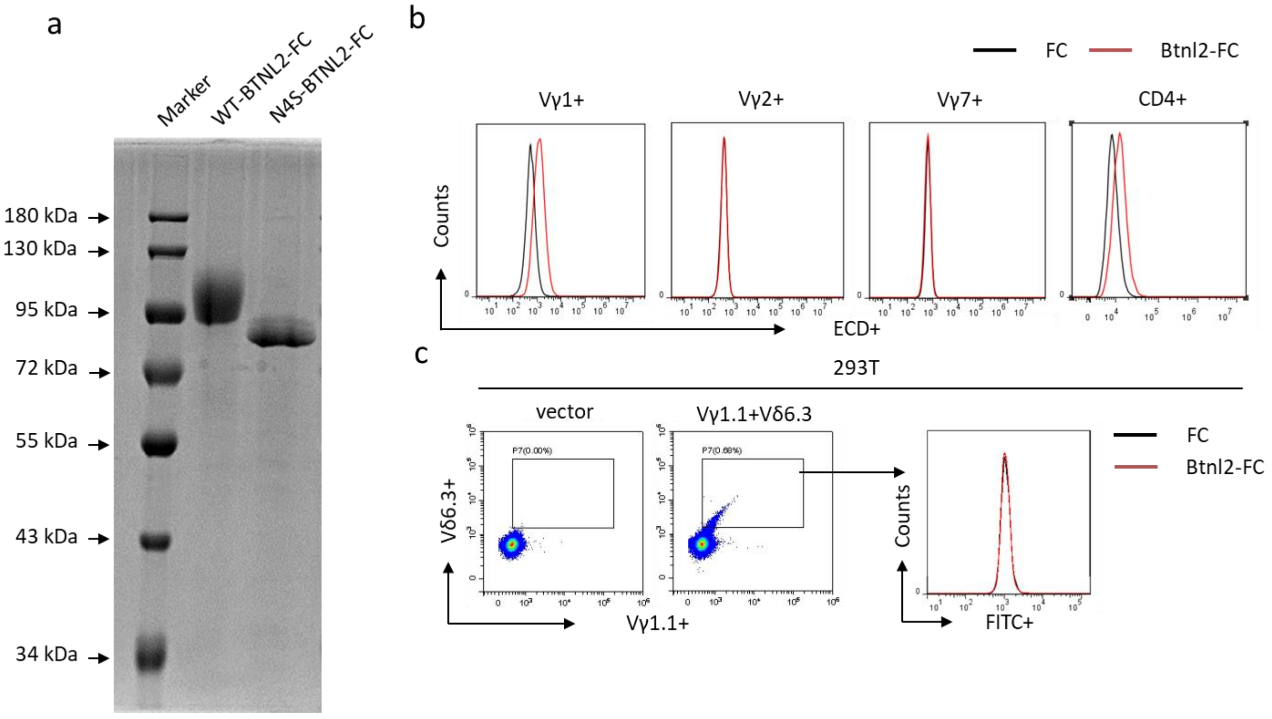

Supplementary Figure 4 BTNL2 binds Vγ1 γδ T cells. (a) WT-BTNL2-FC and N4S-BTNL2-FC recombinant proteins were purified by using 293F cells as described in the Methods. **(b)** FC and BTNL2-FC recombinant proteins were incubated with indicated cells by flow cytometry analysis. **(c)** 293T cells were transfected with vector or Vγ1.1TCR and Vδ6.3TCR, and two days after transfection, cells were incubated with FC and BTNL2-FC recombinant proteins and analyzed by flow cytometry analysis. Data are representative of three independent experiments.

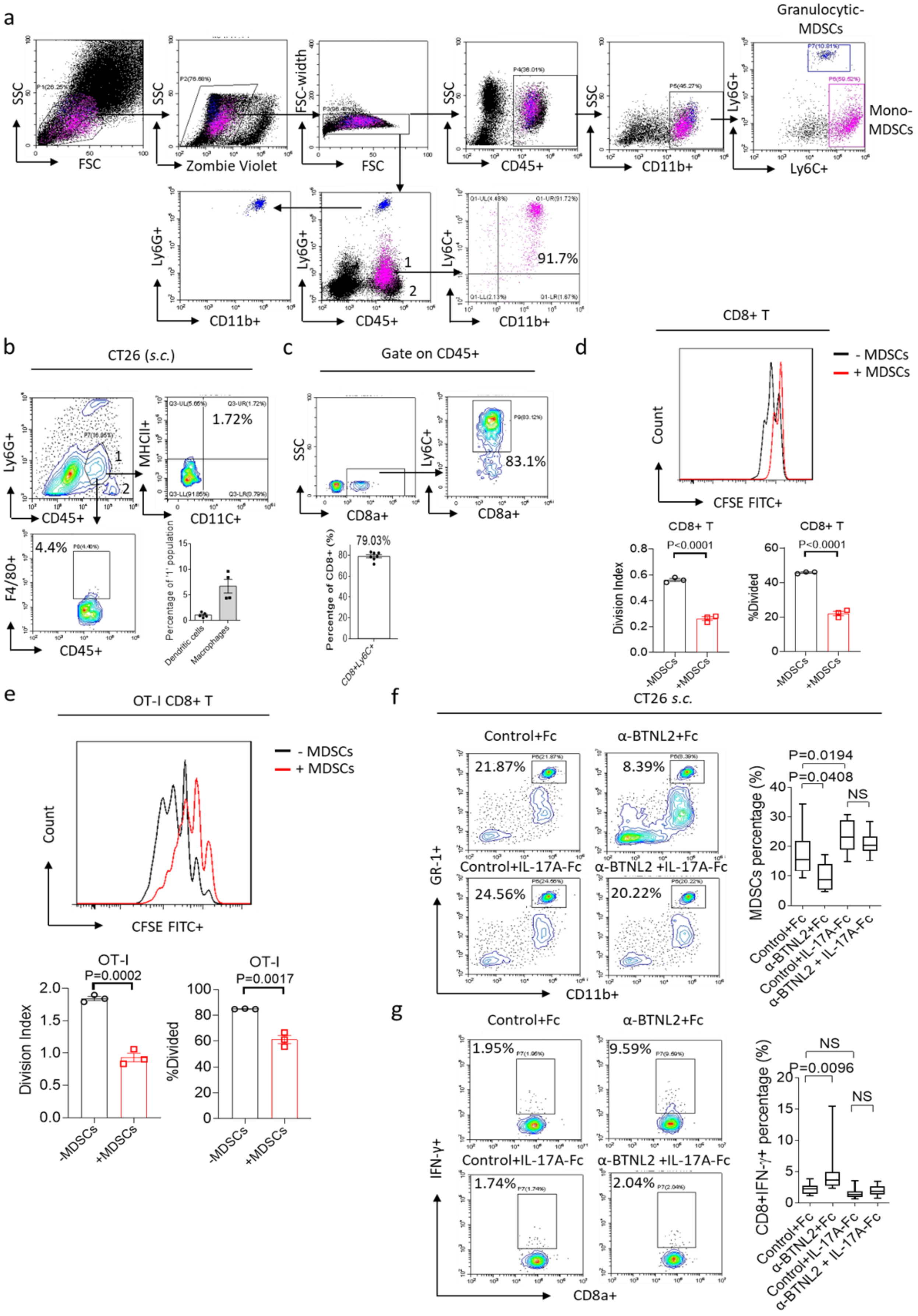
a
SSC
FSC
Zombie Violet
FSC-width
CD45+
CD11b+
Granulocytic-
MDSCs
Ly6G+
Mono-
MDSCs
Ly6C+
91.7%
1
2
b
CT26 (s.c.)
MHCII+
1.72%
CD11C+
F4/80+
4.4%
Percentage of '1' population
Dendritic cells
Macrophages
c
Gate on CD45+
CD8a+
83.1%
Percenge of CD8+ (%)
79.03%
CD8+Ly6C+
d
CD8+ T
- MDSCs
+ MDSCs
Count
CFSE FITC+
Division Index
%Divided
P<0.0001
-MDSCs
+MDSCs
e
OT-I CD8+ T
OT-I
P=0.0002
P=0.0017
f
CT26 s.c.
Control+Fc
α-BTNL2+Fc
21.87%
8.39%
Control+IL-17A-Fc
α-BTNL2 +IL-17A-Fc
24.56%
20.22%
GR-1+
CD11b+
MDSCs percentage (%)
P=0.0194
P=0.0408
NS
α-BTNL2 + IL-17A-Fc
g
1.95%
9.59%
1.74%
2.04%
IFN-γ+
CD8+IFN-γ+ percentage (%)
P=0.0096

Supplementary Figure 5 Tumour-infiltrated 'no. 1' cell population were mainly Mono-MDSCs. **(a-b)** CT26 subcutaneous tumour-infiltrated cell were isolated, followed by flow cytometry analysis as indicated (n=4 for **b**). **(c)** CT26 tumour-infiltrated cell were isolated, followed by flow cytometry analysis. The percentages of CD8+ T cells expressing Ly6C was shown in the lower panel (n=7). **(d)** Flow cytometry-sorted CD8+ T cells were incubated with or without MDSCs (1:1), which were sorted from CT26 tumour-bearing mice. Proliferation was analyzed by CSFE flow cytometry analysis according to the method described in the Methods (n=3, P<0.0001). **(e)** Splenocytes from OT-1 mice were incubated with LLC tumour-sorted MDSCs at 3:1, and stimulated with OVA peptide (100 μg/ml) for three days. Proliferation was analyzed by CSFE flow cytometry analysis according to the method described in the Methods (n=3, P=0.0002 for – MDSC vs +MDSC Division Index, and P=0.0017 for – MDSC vs +MDSC %Division). **(f-g)** Infiltrated cells in CT26 subcutaneous tumour (18 days after tumour implantation) were analyzed by flow cytometry (**f**, n=14, P=0.0408 for Control+Fc vs α-BTNL2+Fc MDSCs percentage, P=0.0194 for Control+Fc vs Control+IL-17A-Fc MDSCs percentage, NS for Control+IL-17A-Fc vs α-BTNL2+IL-17A-Fc MDSCs percentage, and **g**, n=14, P=0.0096 for Control+Fc vs α-BTNL2+Fc $CD8^{+}IFN\text{-}\gamma^{+}$ percentage, NS for Control+Fc vs Control+IL-17A-Fc and Control+IL-17A-Fc vs α-BTNL2+IL-17A-Fc $CD8^{+}IFN\text{-}\gamma^{+}$ percentage). All data are mean±s.e.m. *P<0.05, **P < 0.01, ***P < 0.001, ****P < 0.0001 based on two-sided unpaired T test for **(d-e)** and one-way ANOVA for (**f-g**). Data are representative of three independent experiments for **a-e**, and two independent experiments for **f-g**. The gating strategy for Figure 2a, 2b, 2c, 3a, 3b, 4a, 4b, 4c, 4e, 4f, 4g, 4i and 4j were presented as supplementary figure 5a.

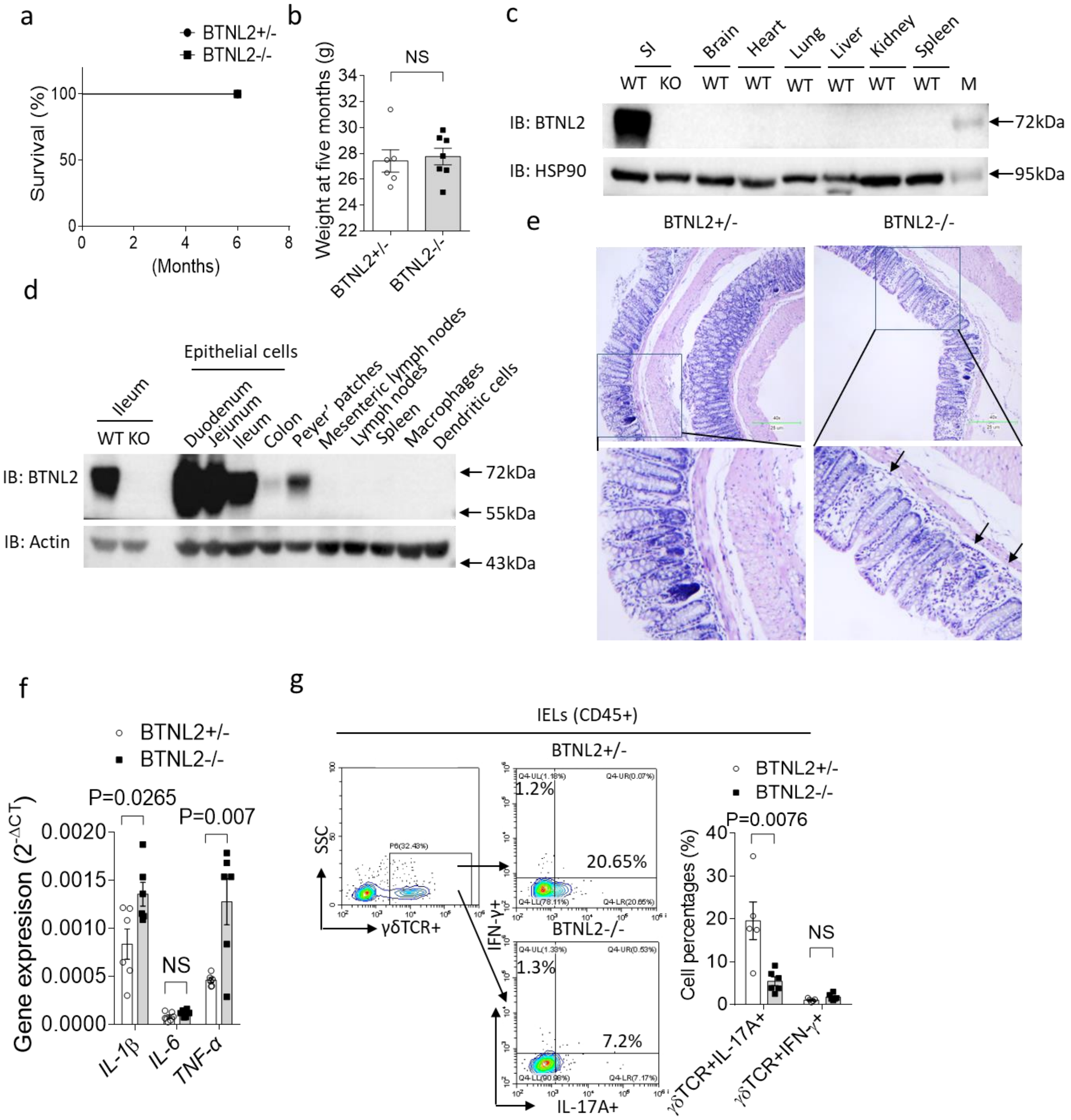
a
BTNL2+/-
BTNL2-/-
Survival (%)
(Months)
b
NS
Weight at five months (g)
BTNL2+/-
BTNL2-/-
c
SI
Brain
Heart
Lung
Liver
Kidney
Spleen
WT KO WT WT WT WT WT WT M
IB: BTNL2
IB: HSP90
72kDa
95kDa
d
Epithelial cells
Ileum
WT KO
Duodenum
Jejunum
Ileum
Colon
Peyer' patches
Mesenteric lymph nodes
Lymph nodes
Spleen
Macrophages
Dendritic cells
IB: BTNL2
IB: Actin
72kDa
55kDa
43kDa
e
BTNL2+/-
BTNL2-/-
f
BTNL2+/-
BTNL2-/-
P=0.0265
P=0.007
NS
Gene expression ($2^{-\Delta CT}$)
IL-1β
IL-6
TNF-α
g
IELs (CD45+)
SSC
γδTCR+
BTNL2+/-
BTNL2-/-
1.2%
20.65%
1.3%
7.2%
IFN-γ+
IL-17A+
P=0.0076
NS
Cell percentages (%)
γδTCR+IL-17A+
γδTCR+IFN-γ+

Supplementary Figure 6 Phenotype analysis of BTNL2-KO mice. (a) Survival curve of littermate control or BTNL2-KO mice was shown. **(b)** Weight of control (n=6, 5 months) or BTNL2-KO mice (n=7, 5 months) was shown (NS for Weight at five months of control vs BTNL2-KO mice). **(c-d)** Western blot analysis of BTNL2 expression in different cells or tissues from littermate control mice or BTNL2-KO mice was shown (1μg/ml of mAb-2 was used for western blot). **(e)** Representative immunohistochemistry images of colon samples from control or BTNL2-KO mice were shown. Arrows indicate the sites of incompetence of crypts. Scale bar=25 μm. **(f)** mRNA was isolated from colonic tissue samples from littermate control mice or BTNL2-KO mice, followed by real-time PCR analysis of indicated genes (n=6 for each group, P=0.0265 for gene expression of *IL-1β*, P=0.007 for gene expression of *TNF-α*, NS for gene expression of *IL-6*). **(g)** Lymphocytes were isolated from indicated tissues, followed by flow cytometry analysis as indicated (n=5 for each group, P=0.0076 for $\gamma\delta TCR^+IL\text{-}17A^+$ cell Percentage, NS for $\gamma\delta TCR^+IFN\text{-}\gamma^+$ cell Percentage). All data are mean±s.e.m. *P<0.05, **P < 0.01, ***P < 0.001, ****P < 0.0001 based on two-tailed unpaired T test for **(b, f-g)**. Data are representative of three independent experiments.

a

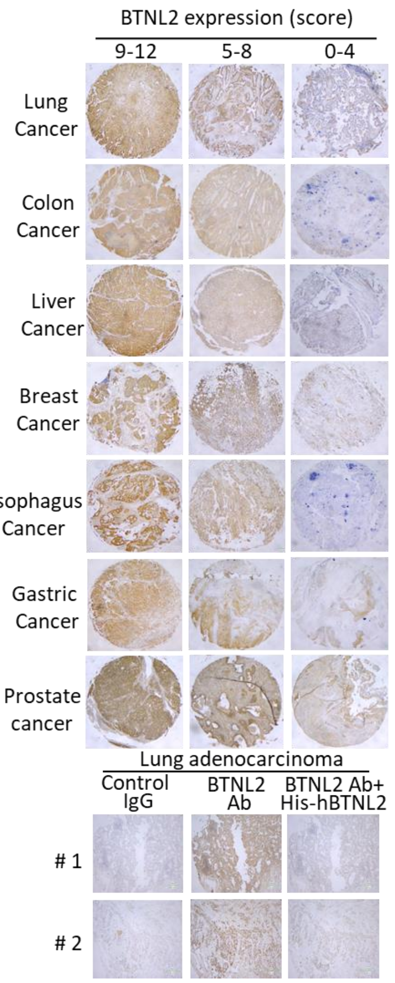

b

Cancer types	Cases with staining intensity		
	9-12	5-8	0-4
Lung adenocarcinoma	28(31%)	35(38%)	28(31%)
Colon adenocarcinoma	36(37%)	45(46%)	17(17%)
Liver cancer	16(40%)	10(25%)	14(35%)
Breast cancer	1(3%)	20(67%)	9(30%)
Esophagus cancer	12(40%)	15(50%)	3(10%)
Gastric cancer	7(18%)	22(55%)	11(27%)
Prostate cancer	8(19%)	27(64%)	7(17%)

c

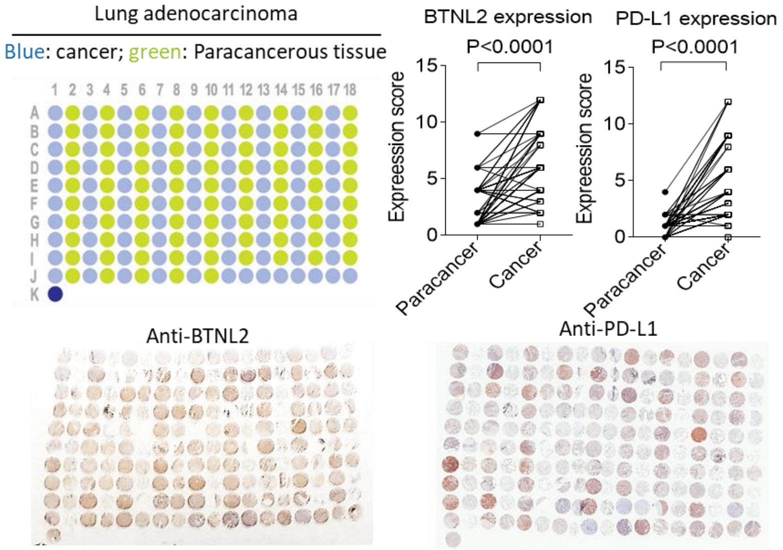

d

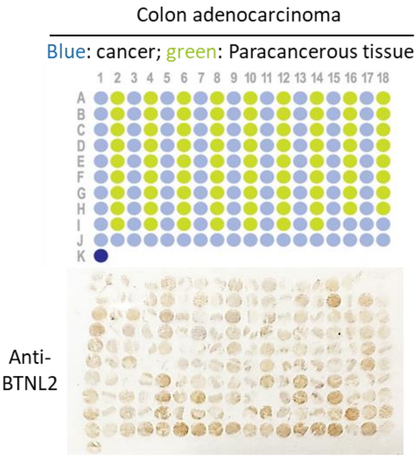

e

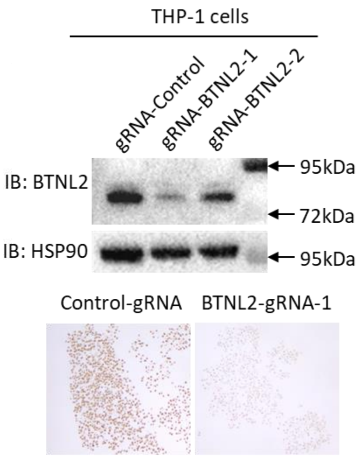

f

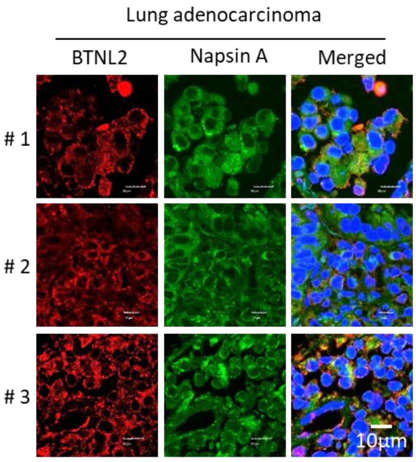

Supplementary Figure 7 Various types of human cancers express BTNL2. (a) Upper panel: representative immunohistochemistry staining of BTNL2 expression in different human cancer types by using cancer tissue chips. The subjects were divided into three groups based on BTNL2 expression scores in the cancers, representing low (scores 1–4), medium (scores 5–8) and high (scores 9–12) expression of BTNL2 according to immunohistochemistry scoring system described in the Materials and Methods. Lower panel: lung adenocarcinoma samples chips was stained with same isotype IgG, anti-BTNL2 antibody or anti-BTNL2 antibody plus His-BTNL2 recombinant protein (10μg/ml) for immunohistochemistry staining. **(b)** BTNL2 expression was scored using the immunohistochemical scores system as described in the Materials and methods, and quantitative analysis of BTNL2 expression in different cancer types were shown. **(c-d)** Overview of immunohistochemistry staining of BTNL2 and PD-L1 by using human lung adenocarcinoma **(c)** and colon adenocarcinoma **(d)** chip samples. Quantification of BTNL2 or PD-L1 expression was shown on the right panel of **(c)** (n=86, P<0.0001 for BTNL2 and PD-L1expression from Paracancer vs Cancer). **(e)** Western blot analysis of BTNL2 in control or BTNL2-gRNA infected THP-1 cells was shown (upper panel). Control or BTNL2-gRNA infected THP-1 cells were analyzed by immunohistochemistry staining by BTNL2 Ab according to the protocol described in the Methods (lower panel). **(f)** Immunofluorescence staining was performed by using lung adenocarcinoma chip samples from three patients with anti-BTNL2 or anti-Napsin A antibody for confocal analysis. Scale bars=10μm. All data are mean±s.e.m. *P<0.05, **P < 0.01, ***P < 0.001, ****P < 0.0001 based on two-tailed unpaired T test for **(c)**. Data are representative of two independent experiments for **(f)**.

Btnl2 mouse QPCR primers	Forward	Reverse
Btnl2 exon2	TAGTCTATCTGGCGTGGCTG	CACGTTAGCAGGGCATCTTC

BTNL2 mice genotyping primers	Forward	Reverse
Mouse *Btnl2*	CGCCCGGCTTATTAAGTCCTTGA	ATGGGAGGTGACAGGAGAAGGCAT
Mouse *Btnl2*	GCATGCTGTGCTGTTACACTG	AGCGTGCACAAGACCTCAAGCT

QPCR primers	Forward	Reverse
Mouse *Btnl2*	ATTACCCTGCAGTCCACGTG	CCACCCATCAGACGTACACC
Mouse *PD-L1*	TATGGCAGCAACGTCACGAT	GGGCATTGACTTTCAGCGTG
mouse *RORC*	CCGCTGAGAGGGCTTCAC	TGCAGGAGTAGGCCACATTACA
mouse *IL-1β*	TGACGGACCCCAAAAGATGA	TCTCCACAGCCACAATGAGT
mouse *TNFα*	CAAAGGGAGAGTGGTCAGGT	ATTGCACCTCAGGGAAGAGT
mouse *IL-6*	GGACCAAGACCATCCAATTC	ACCACAGTGAGGAATGTCCA
mouse *actin*	GGTCATCACTATTGGCAACG	ACGGATGTCAACGTCACACT

BTNL2 KO mice (gRNA sequence)	
gRNA1	AGCTTTCTGATATAGCGTGTGGG
gRNA2	GTGGATGGTACAATTAGCACTGG

gRNA sequence KO cell lines	
gRNA -1 (LLC)	CGCCAGATAGACTATACCGTGGG
gRNA -2 (LLC)	TTTTGTAGATGACTTCAGAGTGG
gRNA -1 (THP-1)	ATAGAAGACTTTAGAGTCATTGG
gRNA -2 (THP-1)	CCGGCCAGGATAGGATGAGCAGG

Supplementary Table 1 QPCR and genotyping primer sequence were shown.
