# Supplementary material for: Cancer cell-expressed BTNL2 facilitates tumour immune escape via engagement with IL-17A-producing γδ T cells
Source: Nat Commun. 2022 Jan 11;13:231. doi: 10.1038/s41467-021-27936-8 (PMC8752682; doi:10.1038/s41467-021-27936-8)
Supplement: Supplementary file 3 — Reporting Summary [file 41467_2021_27936_MOESM3_ESM.pdf]

nature portfolio

Corresponding author(s): Chenhui Wang

Last updated by author(s): Nov 6, 2021

Reporting Summary

Nature Portfolio wishes to improve the reproducibility of the work that we publish. This form provides structure for consistency and transparency in reporting. For further information on Nature Portfolio policies, see our Editorial Policies and the Editorial Policy Checklist.

Statistics

For all statistical analyses, confirm that the following items are present in the figure legend, table legend, main text, or Methods section.

n/a	Confirmed	
☐	☒	The exact sample size (*n*) for each experimental group/condition, given as a discrete number and unit of measurement
☐	☒	A statement on whether measurements were taken from distinct samples or whether the same sample was measured repeatedly
☐	☒	The statistical test(s) used AND whether they are one- or two-sided<br>*Only common tests should be described solely by name; describe more complex techniques in the Methods section.*
☒	☐	A description of all covariates tested
☐	☒	A description of any assumptions or corrections, such as tests of normality and adjustment for multiple comparisons
☐	☒	A full description of the statistical parameters including central tendency (e.g. means) or other basic estimates (e.g. regression coefficient) AND variation (e.g. standard deviation) or associated estimates of uncertainty (e.g. confidence intervals)
☐	☒	For null hypothesis testing, the test statistic (e.g. *F*, *t*, *r*) with confidence intervals, effect sizes, degrees of freedom and *P* value noted<br>*Give P values as exact values whenever suitable.*
☒	☐	For Bayesian analysis, information on the choice of priors and Markov chain Monte Carlo settings
☒	☐	For hierarchical and complex designs, identification of the appropriate level for tests and full reporting of outcomes
☐	☒	Estimates of effect sizes (e.g. Cohen's *d*, Pearson's *r*), indicating how they were calculated

Our web collection on statistics for biologists contains articles on many of the points above.

Software and code

Policy information about availability of computer code

Data collection	No software was used for the data collection.
Data analysis	Graphpad Prism Version 8 was used to analyze the data. CytExpert 2.3 and Flowjo 7.6.1 was used to analyze the FACS data. FV31S-SW was used to analyze the Immunofluorescence data.

For manuscripts utilizing custom algorithms or software that are central to the research but not yet described in published literature, software must be made available to editors and reviewers. We strongly encourage code deposition in a community repository (e.g. GitHub). See the Nature Portfolio guidelines for submitting code & software for further information.

Data

Policy information about availability of data

All manuscripts must include a data availability statement. This statement should provide the following information, where applicable:

- Accession codes, unique identifiers, or web links for publicly available datasets
- A description of any restrictions on data availability
- For clinical datasets or third party data, please ensure that the statement adheres to our policy

The source data underlying Fig. 1-6, Supplementary Fig. 1-7 is provided as a Source Data file. Other data that support the study are available from the corresponding author (wangchenhui@hust.edu.cn). There are no accession codes, unique identiifers and web linkers which were generated by this study.

Field-specific reporting

Please select the one below that is the best fit for your research. If you are not sure, read the appropriate sections before making your selection.

- [x] Life sciences
- [ ] Behavioural & social sciences
- [ ] Ecological, evolutionary & environmental sciences

For a reference copy of the document with all sections, see nature.com/documents/nr-reporting-summary-flat.pdf

Life sciences study design

All studies must disclose on these points even when the disclosure is negative.

Sample size	Sample sizes are provided in each figure legend, and we chosen the sample size based on the previous studies:<br>1) Nat Commun. 2020 Apr 20;11(1):1913;<br>2) Nat Commun. 2018 Jul 16;9(1):2745;<br>3) Nat Immunol. 2016 May;17(5):583-92.
Data exclusions	No data were excluded.
Replication	All data presented has been replicated. Most graphs display the collective data from several independent experiments and the number of replicate experiments included are given in the figure legends.
Randomization	Genetic mouse experiment: Different genotypes from the same litter were compared. Wild-type mice were randomly divided into control treatment or treatment groups. Other experiments are not relevant to the application of randomization.
Blinding	During assessment of the tumor size and mice survival, the assessor did not know the genotypes or treatments the mice received. For the flow cytometry experiments of Figure 2a, 2b, 2c, 4a, 4b, 4c, 4d, 4e, 4f, 4g, 4j, 4k and 5e, the person who analyzed the data by flow cytometry was blinded to the genotypes or treatments.

Reporting for specific materials, systems and methods

We require information from authors about some types of materials, experimental systems and methods used in many studies. Here, indicate whether each material, system or method listed is relevant to your study. If you are not sure if a list item applies to your research, read the appropriate section before selecting a response.

Materials & experimental systems

n/a	Involved in the study	
☐	☒	Antibodies
☐	☒	Eukaryotic cell lines
☒	☐	Palaeontology and archaeology
☐	☒	Animals and other organisms
☐	☒	Human research participants
☒	☐	Clinical data
☒	☐	Dual use research of concern

Methods

n/a	Involved in the study	
☒	☐	ChIP-seq
☐	☒	Flow cytometry
☒	☐	MRI-based neuroimaging

Antibodies

Antibodies used	1. A rat-anti-mouse BTNL2 monoclonal antibody (clone: mAb-2) was made by Atagenix company (this is an antibody customized from the Atagenix company, so it doesn't have a catalog number). Isotype control rat IgG1 antibody was bought from R&D Systems (Clone 43414R, cat no. MAB005R).<br>2. Anti-human BTNL2 pAb for western blot was bought from Proteintech (1:1000, cat no. 25110-1-AP). Anti-BTNL2 polyclonal antibody for IHC and IF was bought from Sigma (1:100, cat no. HPA039844). Anti-α-Tubulin (1:1000, clone 11H10, cat no. 2125) and Anti-PD-L1 antibodies for IHC were bought from CST (1:200, clone (E1L3N®) XP®, cat no. 13684). Anti-human PD-L1 antibody for western blot was bought from Proteintech (1:2000, clone 2B11D11, cat no. 66248-1-Ig). Anti-Na/K ATPase (1:5000, cat no. 14418-1-AP) polyclonal antibody were bought from Proteintech. Anti-β-Actin (1:1000, clone C4, cat no. sc-47778) and anti-HSP90 (1:1000, clone F-8, cat no. sc-13119) antibodies were bought from SANT CRUZ. Anti-Napsin A antibody was bought from Proteintech (1:500, clone 2D12A2, cat no. 60259-1-Ig).<br>3. Anti-PD-1 (clone RMP1-14, cat no. BE0146), anti-Vγ1 TCR (clone 2.11, cat no. BE0257), anti-IL-17A (clone 17F3, cat no. BE0173), anti-CD8a (clone 2.43, cat no. BE0061), anti-CD11b (clone M1/70, cat no. BE0007), Polyclonal Armenian hamster IgG (cat no. BE0091), anti-keyhole limpet hemocyanin (clone LTF-2, cat no. BE0090) for the neutralization were bought from BioXcell.<br>4. Flow antibodies of anti-CD4 (clone RM4-5, cat no. 100510), anti-CD8a (clone 53-6.7, cat no. 100708), anti-IL-17A (clone of TC11-18H10.1, cat no. 506904), anti-IFN-γ (clone XMG1.2, cat no. 505810), anti-IL-17A (clone of TC11-18H10.1, cat no. 506916), anti-IFN-γ (clone XMG1.2, cat no. 505806), anti-IFN-γ (clone XMG1.2, cat no. 505829), anti-γδ TCR (clone UC7-13D5, cat no. 107504), anti-γδ TCR (clone UC7-13D5, cat no. 107512), anti-γδ TCR (clone GL3, cat no. 118116), anti-γδ TCR (clone GL3, cat no. 118124), anti-Vγ1.1TCR (2.11, cat no. 141103), anti-Vγ4 TCR (clone UC3-10A6, cat no. 137703), anti-CD45 (clone of 30-F11, cat no. 103108), anti-

CD45 (clone of 30-F11, cat no.103116), anti-GR-1 (clone RB6-8C5, cat no. 108407), anti-Ly6G (clone 1A8, cat no. 127608), anti-F4/80 (clone BM8, cat no. 123115), anti-CD11b (clone M1/70, cat no. 101212), anti-CD11b (clone M1/70, cat no. 101207), anti-Ly6C (clone HK1.4, cat no. 128017), anti-CD11c (clone N418, cat no. 117310), anti-CD16/32 (clone 93, cat no. 101302), anti-Asialo GM1 (clone Poly21460, cat no. 146007) anti-CD3ε (clone 17A2, cat no. 100236), anti-I-A/I-E (clone M5/114.15.2, cat no. 107607) and anti-CD27 (clone LG.3A10, cat no. 124211) were bought from Biolegend. True-Nuclear™ Mouse Treg Flow™ Kit was bought from Biolegend (cat no. 320029). Antibodies of anti-human CD3ε (clone HIT3a, cat no. 300306), anti-human γδ TCR (B1, cat no. 331222), anti-human IL-17A (clone BL168, cat no. 512306) and anti-human IFN-γ (clone B27, cat no. 502512) were bought from Biolegend. Anti-mouse Vγ7 TCR antibody was kindly provided by Dr. Pablo Pereira at Institut Pasteur in France. Antibodies of anti-mouse CD3ε (clone 145-2C11, cat no.100340) and anti-mouse CD28(clone 37.51, cat no.102116) were bought from Biolegend.

Validation

Anti-BTNL2 mAb-2 is working for blocking, western blot and flow cytometry analysis for mouse BTNL2, as shown in supplementary figure1a, 1c, 1d, 1e, 2a, 2d, 2g, 6c, 6d, and we didn't test whether this mAb work for human BTNL2 or not.

All the commercial antibodies were validated by the suppliers, and the validation information can be found on the company's websites.
Anti-human BTNL2 polyclonal antibody (Proteintech , cat no. 25110-1-AP).
Validation: https://www.ptgcn.com/products/BTNL2-Antibody-25110-1-AP.htm

Anti-BTNL2 polyclonal antibody (Sigma, cat no. HPA039844).
Validation: PMID: 25489103(IHC)

Anti-α-Tubulin (CST, Cat No: 2125)
Validation: https://www.cellsignal.cn/products/primary-antibodies/a-tubulin-11h10-rabbit-mab/2125?site-search-type=Products&N=4294956287&Ntt=2125&fromPage=plp&_requestid=3237937

Anti-PD-L1 (CST, cat no. 13684)
Validation: PMID: 34178637(IHC)

Anti-human PD-L1 antibody (Proteintech, cat no. 66248-1-Ig).
Validation: PMID: 30979816(WB)

Anti-Na/K ATPase : (Proteintech, Cat No: 14418-1-AP)
Validation: PMID: 32574956 (WB)

Anti-β-Actin (SANT CRUZ, cat no. sc-47778)
Validation: https://www.scbt.com/p/beta-actin-antibody-c4?requestFrom=search

Anti-HSP90 (SANT CRUZ, cat no. sc-13119)
Validation: https://www.scbt.com/p/hsp-90alpha-beta-antibody-f-8?requestFrom=search

Anti-Napsin A (Proteintech , cat no. 60259-2-Ig)
Validation: https://www.ptgcn.com/products/NAPSA-Antibody-60259-2-Ig.htm

Eukaryotic cell lines

Policy information about cell lines

Cell line source(s)	HEK293T, B16F10,LLC, MC38, CT26, A20,4T1,THP-1 and 293F cell lines were bought from ATCC.
Authentication	Cell lines were authenticated by short tandem repeat profiling.
Mycoplasma contamination	All cell lines are negative for mycoplasma.
Commonly misidentified lines (See ICLAC register)	No commonly misidentified cell lines were used in this study.

Animals and other organisms

Policy information about studies involving animals; ARRIVE guidelines recommended for reporting animal research

Laboratory animals	BTNL2-KO mice were made by Cyagen Biosciences Inc by Crispr-cas9. BTNL2-KO mice were C57BL/6 background. Six to eight weeks old female C57BL/6 and BALB/c mice were obtained from Beijing Vital River Laboratory Animal Technology Co., Ltd. OT-1 transgenic mice were kindly provided by Prof. Zhengfan Jiang at Peking University and six to eight weeks old male OT-1 transgenic mice were used in our experiments. These mice were housed in SPF condition, the ambient temperature is between 20-25°C, the humidity is between 40-70%, and the environmental light/dark cycle is 12h light, 12h dark. Laboratory animals used are described in the Method section.
Wild animals	The study did not involve wild animals.
Field-collected samples	The study did not involve samples collected from the field.

Ethics oversight	All animals were used according to protocols approved by the Institutional Animal Care and Use Committee of Tongji Medical College, Huazhong University of Science & Technology.

Note that full information on the approval of the study protocol must also be provided in the manuscript.

Human research participants

Policy information about studies involving human research participants

Population characteristics	Lung cancer samples and paired para-cancerous tissue samples were obtained from patients with lung adenocarcinoma who underwent surgical resection at the Department of Thoracic Surgery, Renmin Hospital of Wuhan University. Live cancer samples and paired para-cancerous tissue samples were obtained from patients with hepatocellular carcinoma who underwent surgical resection at the department of Hepatic Surgery Center, Tongji Hospital, Tongji Medical College of Huazhong University of Science and Technology. When we collected cancer samples, we did not consider the factors such as patient's gender, age, and so on.
Recruitment	When we collected cancer samples, we did not consider the factors such as patients' gender, age, and so on.
Ethics oversight	The protocols were approved by the Review Board of the Renmin Hospital of Wuhan University (Approval No: WDRY2019-K063), Tongji Medical College of Huazhong University of Science and Technology (Approval No: S1231).

Note that full information on the approval of the study protocol must also be provided in the manuscript.

Flow Cytometry

Plots

Confirm that:

- [x] The axis labels state the marker and fluorochrome used (e.g. CD4-FITC).
- [x] The axis scales are clearly visible. Include numbers along axes only for bottom left plot of group (a 'group' is an analysis of identical markers).
- [x] All plots are contour plots with outliers or pseudocolor plots.
- [x] A numerical value for number of cells or percentage (with statistics) is provided.

Methodology

Sample preparation	Mice tumors were collected, dissociated mechanically, digested with 2 mg/ml Collagenase Ⅳ (sigma) and 0.2 mg/ml DNase I (Biofroxx) in serum-free DMEM medium at 37°C. After 40 min, enzyme activity was neutralized by addition of cold RPMI-1640/10% FBS and tissues were passed through 70μM cell strainers (Biologix group Limited) and single cell suspensions in T cell culture medium (RPMI-1640, 10% FBS, 100 IU/ml penicillin, 100 μg/ml streptomycin, 0.5% β-mercaptoethanol) were stimulated with Cell Activation Cocktail (with Brefeldin A) for 4 hours (for intracellular staining). After stimulation, cells were incubated with anti-CD16/CD32 (Biolegend) or Human TruStain FcX™ before staining with fluorochrome-conjugated monoclonal antibodies. Cell surface staining was done for 30 min at 4°C. Intracellular staining was done using a fixation/permeabilization kit (Biolegend). Zombie Violet Fixable Viability kit (1:400; Biolegend) was added to exclude dead cells.<br>Spleen were isolated from mice, and were smashed using a strainer (red blood cells were lysised) to get the single cell solution for flow cytometry staining.<br>The small intestine was opened longitudinally, freed of Peyer's patches, and washed in serum-free RPMI 1640 medium. Intestinal tissues were cut into 5 mm fragment, transferred to a 50 ml plastic conical centrifuge tube (Jet Biofil) and incubated for 30 min in RPMI 1640 medium supplemented with 2% penicillin/streptomycin, 10% Fetal Bovine Serum, 1mM DTT and 1mM EDTA on 37°C incubator. Then the tissues were passed through a 70μM strainer to remove tissue pieces and centrifuged on a 40%/80% Percoll density gradient at 2500 rpm for 25 minutes. IEL were harvested from the 40% to 80% Percoll interface for flow cytometry staining.
Instrument	Beckman Coulter CYTOFLEX flow cytometry system
Software	CytExpert 2.3 and Flowjo 7.6.1
Cell population abundance	For tumor analysis, at least 50, 000 CD45+ cells were analyzed. For spleen cell analysis at least 10, 000 γδ T+ and 20,000 CD4+ T cells were analyzed. For the IEL, all of the cells isolated from small intestine were analyzed .
Gating strategy	The gating strategy for Figure 2a, 2b, 2c, 3a, 3b, 4a, 4b, 4c, 4e, 4f, 4g, 4i and 4j were presented as supplementary figure 5a. The full gating strategy was also presented in the Figure 3c and 3d.

- [x] Tick this box to confirm that a figure exemplifying the gating strategy is provided in the Supplementary Information.
